# Cigarette and E-Cigarette Use and Smoking Cessation Practices among Physicians in Poland

**DOI:** 10.3390/ijerph16193595

**Published:** 2019-09-25

**Authors:** Mateusz Jankowski, Dorota Kaleta, Wojciech Stefan Zgliczyński, Justyna Grudziąż-Sękowska, Iwona Wrześniewska-Wal, Mariusz Gujski, Waldemar Wierzba, Jarosław Pinkas

**Affiliations:** 1 School of Public Health, Centre of Postgraduate Medical Education, Kleczewska 61/63, 01-826 Warsaw, Poland; 2 Department of Hygiene and Epidemiology, Medical University of Lodz, Zeligowskiego 7/9 Str, 90-752 Łódź, Poland; 3 Department of the Prevention of Environmental Hazards and Allergology, Medical University of Warsaw, Banacha 1a Str, 02-091 Warsaw, Poland; 4 UHE Satellite Campus in Warsaw, University of Humanities and Economics in Łódź, Felińskiego 15 Str, 01-513 Warsaw, Poland

**Keywords:** tobacco use, smoking, electronic cigarettes, physicians, smoking cessation, addiction

## Abstract

Physicians play a key role in combating tobacco use. This study aims to evaluate the knowledge, attitude, and behaviors toward smoking cessation and vaping cessation interventions among physicians in Poland; to identify factors shaping physicians’ behaviors toward smoking and vaping cessation interventions; and to assess differences in the tobacco cessation interventions recommended for cigarette smokers and users of electronic cigarettes (e-cigarettes). A questionnaire-based survey was conducted in 2018 among physicians attending mandatory public health training courses delivered at the School of Public Health, Centre of Postgraduate Medical Education, Warsaw, Poland. The questionnaire included 25 questions related to tobacco product use and smoking cessation interventions. Data were obtained from 423 physicians (64.3% female; mean age 32.0 ± 5.8 years) with response rate of 84.6%. Current cigarette smoking was declared by 7.8% of participants; 1.9% of participants were e-cigarette users and 1.9% used heated tobacco. Smoking cessation interventions were offered more often to patients who smoked cigarettes than those who used e-cigarettes (*p* < 0.001). Physicians’ behaviors toward smoking cessation and vaping cessation interventions were associated with (*p* < 0.05) physicians’ smoking status and self-declared knowledge about smoking cessation methods. Among physicians in Poland, discussion of smoking cessation was not common behavior and limited mainly to identification of smoking status.

## 1. Introduction

Tobacco use is a leading cause of preventable death [1,2]. To fight the global tobacco epidemic, a number of tobacco control initiatives were taken at local and international levels [3,4,5,6]. Successful tobacco control policy includes tobacco taxation; bans on smoking in public places and elimination of environmental tobacco smoke; restrictions on tobacco advertising, promotion, and sponsorship; effective packaging and labeling laws; and warnings about the dangers of tobacco use [6,7,8]. Education and public awareness about the effects of tobacco consumption, as well as nationwide smoking cessation interventions, are crucial to reduce the demand for tobacco [9].

Physicians play a key role in combating tobacco use and reducing the prevalence of tobacco-related diseases [10,11,12]. Even though a relatively small degree of efficacy (between 1 and 3% [10]) is achieved from brief advice-based interventions from physicians, the cumulative effect is measurably evident across populations. Routinely offering smoking cessation interventions to large numbers of physicians may substantially reduce smoking rates [10,12,13]. In many countries, national guidelines for smoking cessation were developed to prepare physicians to systematically identify their smoking patients and offer them smoking cessation advice [14,15,16,17,18]. Smokers are most often identified and advised in a primary care setting [19]. A review of the national guidelines for smoking cessation from 22 countries revealed almost universal agreement regarding the need to identify smokers and offer some form of advice to quit as well as to offer behavioral and pharmacological support to quit [15]. In Poland, smoking cessation guidelines recommend healthcare professionals (especially general practitioners) systematically identify smokers and provide a minimal intervention based on the “Five As” (Ask, Advise, Assess, Assist, Arrange) [16,17]. All medical students in Poland are trained to deliver smoking cessation intervention (Five As) as a part of medical training [16]. Moreover, there is a free-of-charge postgraduate training course addressed to all physicians, who want to offer professional smoking cessation counseling (pharmacotherapy and behavioral interventions for smoking cessation) in their medical practice [20]. Between 2016 and 2020 the training program will cover 2500 healthcare professionals including doctors and nurses [21].

Patients’ growing interest in electronic cigarettes (e-cigarettes) and heated tobacco has led some medical organizations to prepare position statements and update their guidelines to include information regarding e-cigarettes (vaping cessation guidelines) and novel tobacco products [18,22,23,24]. The national smoking cessation guidelines do not include e-cigarettes and minimal intervention “Five As” does not apply to e-cigarette users. However, according to Polish law, e-cigarettes are considered equivalent to conventional tobacco cigarettes [25]. Moreover, a recent study showed that most of physicians in Poland perceive e-cigarettes as harmful (96%) and addictive (97%) [26], which may suggest a similar physicians’ behaviors toward cigarette or e-cigarette use by the patients. 

Data on smoking cessation and vaping cessation interventions provided by physicians in Central and Eastern Europe are very limited [27]. Moreover, there is a lack of study assessing the smoking cessation and vaping cessation practices provided by physicians in Poland. However, studies performed among physicians from Europe and the United States (US) showed that physicians there do not routinely provide smoking cessation intervention to their patients [28,29,30]. 

Physicians’ engagement in smoking cessation interventions is influenced by personal skill and knowledge about smoking cessation methods and organizational factors such as time required to brief patients on smoking cessation interventions [30,31]. Some studies suggest that physicians’ smoking status can influence attitudes toward smoking cessation interventions offered to patients [27,32,33,34]. However, most consider only tobacco use (without the potential impact of other nicotine-containing products such as e-cigarettes) as a potential factor shaping physicians’ attitudes toward smoking cessation intervention [27,32]. Moreover, there are only limited studies exploring how physicians approach smoking cessation interventions for patients who use e-cigarettes [35,36]. Therefore, the objectives of this study were to (1) evaluate the knowledge, attitude, and behaviors toward smoking cessation and vaping cessation interventions among physicians in Poland; (2) identify factors shaping physicians’ attitudes toward smoking cessation and vaping cessation interventions; and (3) assess differences in tobacco cessation interventions for cigarette smokers and e-cigarette users in daily medical practice.

## 2. Materials and Methods

### 2.1. Participants

A questionnaire-based survey was conducted between September and December 2018. Within Poland, each of 25,000 physicians undergoing specialty certification must attend a public-health training course within five to six years of beginning their specialist training. We approached physicians attending the autumn 2018 training courses (a total of eight different courses). All 500 physicians attending these public health training courses delivered at the School of Public Health, Centre of Postgraduate Medical Education, Warsaw, Poland, during this period were eligible to take the survey. The participants represented different regions and healthcare institutions from across Poland.

### 2.2. Study Questionnaire 

The research tool was an original questionnaire developed for the purpose of this study. In preparation of the questionnaire, we analyzed the previously published studies on the smoking cessation practices among healthcare professionals [27,37,38]. The questionnaire included 25 questions related to tobacco-product use and smoking cessation interventions in daily medical practice. Questions also addressed background information including age, sex, medical education level, specialty, years of professional experience, and medical practice characteristics (practice type and location). Depending on the medical education level, participants were assigned to either the residents or specialists group. A self-declared field of medical practice was used to assign subjects to surgical or nonsurgical specialties. Based on declared place of primary practice, participants were assigned to a hospital or an ambulatory practitioners group.

E-cigarette or heated tobacco product awareness was defined according to answers of the following questions: “Are you aware of electronic cigarettes (e-cigarettes)?” and “Are you aware of heated tobacco products?” (“Yes”/“No”). 

Questions to determine if a physician had ever used cigarette, e-cigarette, or heated tobacco products included “Have you ever smoked/tried a combustible cigarette?”, “Have you ever used/tried an e-cigarette?” and “Have you ever used/tried heated tobacco products?” Current smoking status was based on the questions: “Do you currently smoke combustible cigarettes?”, “Do you currently use e-cigarettes?” and “Do you currently use heated tobacco products?”, each with two possible answers (“Yes” and “No”).

Contact with smoking patients was defined by answers to the following question: “Do you have contact with a patient who smokes or uses e-cigarettes in a daily medical practice?” ("Yes”/“No”). 

Moreover, questions regarding smoking cessation interventions were addressed. Self-reported knowledge about smoking cessation methods was based on the question, “How do you assess your state of knowledge on anti-smoking counseling and methods of smoking cessation?”, with five possible answers: “very good”, “rather good”, “moderate”, “rather bad”, and “very bad”. For the purpose of regression analyses, three categories were developed (very good or rather good vs. moderate vs. rather bad or very bad. 

To evaluate the treatment of a smoking or vaping patient in physicians’ daily medical practice, a 4-point response scale was formulated to evaluate extent of patients’ cigarette smoking as well as e-cigarette use. These points included (1) taking a smoking history, (2) documentation of smoking status in the health records, (3) minimal intervention on smoking cessation, and (4) referring to anti-smoking counseling. All four statements measured a range of activity on the 4-point response scale: 1 = “yes, every case”, 2 = “yes, in more than half of the cases”, 3 = “yes, in fewer than half of the cases”, and 4 = “no, never”. In the regression analyses two first responses were considered as positive whereas 3 and 4 as negative one.

Originally, the questionnaire was developed in Polish. Repeatability of the prepared questionnaire was assessed. A group of 14 physicians completed the questionnaire twice, five days apart. Questionnaires and the form of distribution in both samples were identical. Depending on the question, the kappa coefficient for the critical questions ranged from 0.88 to 0.96.

Participation in the study was voluntary and anonymous. Informed verbal consent was obtained from all individual participants. All procedures performed in studies involving human participants were in accordance with the ethical standards of the institutional and/or national research committee and with the 1964 Helsinki Declaration and its later amendments or comparable ethical standards. According to the current guidelines of Ethical Review Board at the Centre of Postgraduate Medical Education, Warsaw, Poland, an anonymous, questionnaire-based cross-sectional study does not require separate consent.

### 2.3. Statistical Analysis 

The data were analyzed with Statistica 12 Software (TIBCO Software Inc., Palo Alto, CA, USA) and SPSS version 25 (IBM, Armonk, NY, USA). Normality of distribution of continuous variables was assessed by the Shapiro–Wilk test. The distribution of categorical variables was shown by frequencies and proportions (with 95% confidence interval—95%CI). A chi-square test was used to compare categorical variables. Statistical inference was based on the criterion *p* < 0.05.

The logistic regression analyses were applied to identify the predictors of physicians’ behaviors toward cigarette or e-cigarette use by the patients. Separate analyses, for each physicians’ behavior toward cigarette smoking and each behavior toward e-cigarette use by the patients, were performed. Each behavior, including (1) smoking status identification, (2) smoking status documentation, (3) minimal intervention, and (4) referral to smoking cessation clinics, was considered separately as dependent variable in the model. The socio-demographic characteristics (gender, age), smoking/e-cigarette use/heated tobacco use status, self-reported knowledge of smoking cessation counseling, and characteristics related to medical practice (medical education level, specialty, years of professional experience, medical type, and location) were considered as independent variables. In univariate logistic regression analyses all variables were considered separately. Multivariate logistic regression analyses included all of the variables significantly associated with the physicians’ behaviors toward cigarette or e-cigarette use by the patients in any of the univariate models (*p* < 0.05).

## 3. Results

Completed questionnaires were obtained from 423 physicians (64.3% female; mean age 32.0 ± 5.8 years), with a response rate of 84.6%. There were no age differences between males and females (*p* > 0.05). The vast majority of participants were physicians in training (85.3%) and 72.5% of all were trained in nonsurgical specialties. Hospital as a place of primary employment was declared by 88.4% of participants (Table 1). More than half of the physicians (53%) practiced in cities with 500,000 residents or more (Table 1).

### 3.1. Physicians’ Smoking Status

The predominant group of participants (71.2%) had ever smoked a combustible cigarette; the highest prevalence of ever cigarette use observed among males (78.8%) and physicians in training (73.7%). Females and specialists reported less ever use (66.9%; *p* = 0.01, and 56.5%; *p* = 0.006, respectively). Current cigarette smoking was declared by 7.8% of participants, with no statistically significant (*p* > 0.05) differences depending on sex, medical education level, and specialty (Table 2). 

Almost all of the physicians were aware of e-cigarettes (99.8%) and 42.6% declared to become aware of heated tobacco products. Men were more (*p* < 0.001) aware of heated tobacco than women as well as physicians trained in surgical specialties than nonsurgical specialists (*p* = 0.01). Among respondents, 22.2% had ever tried an e-cigarette (even one puff) and 1.9% were current e-cigarette users (Table 2). The proportion of physicians who had ever used e-cigarettes significantly differed (*p* < 0.05) depending on sex, medical education level, and specialty. Ever heated-tobacco use was declared by 8.5% of respondents, wherein males more often than females tried heated tobacco (13.3% vs. 5.9%; *p* = 0.01). Smoking status did not differ (*p* > 0.05) between physicians primarily employed in hospital or ambulatory and practice location. The prevalence of cigarette, e-cigarette, and heated tobacco use is presented in Table 2.

### 3.2. Smoking Cessation Knowledge

More than half of respondents (53.9%) declared a moderate level of knowledge about smoking cessation interventions, slightly more than one quarter (25.3%) defined their knowledge of smoking cessation as “somewhat strong” and 5.3% of physicians declared strong knowledge of smoking cessation interventions. A little smoking cessation knowledge was declared by 14.2% of participants, whereas lack of smoking cessation knowledge was declared by 1.2% of physicians. The majority of the physicians (78.0%) declared that they have contact with a smoking patient in a daily medical practice. Among the 93 physicians who declared that they do not have contact with smoking patients, 50 were pediatricians, 13 were trained in general surgery or orthopedics, eight were radiologists, and 22 represented 11 additional specialties. 

### 3.3. Physicians’ Behaviors toward Cigarette or E-Cigarette Use by the Patients

There were statistically significant differences (*p* < 0.05) in tobacco cessation interventions for cigarette smokers and e-cigarette users (Table 3). Tobacco cessation interventions were more often offered to patients who smoked cigarettes than to those who used e-cigarettes (Table 3).

The results of univariate and multivariate logistic regression analyses to identify the variables influencing physicians’ behaviors toward cigarette or e-cigarette use by the patients are presented in Table 4 and Table 5. In the multivariate model, physicians who declared a higher level of knowledge of smoking cessation interventions (very good or rather good) had a higher chance of identifying patients’ smoking and e-cigarette use status (OR = 9.8; *p* < 0.001; OR = 2.4; *p* <0.05, respectively), noting smoking and e-cigarette use status in the medical documentation (OR = 6.6; *p* < 0.01; OR = 2.4; *p* < 0.05, respectively), performing minimal intervention on smoking cessation (OR = 3.3; *p* < 0.01; OR = 2.7; *p* < 0.05, respectively), and referring smokers or e-cigarette users to smoking cessation clinics (OR = 4.4; *p* < 0.05; OR = 8.0; *p* <0.05, respectively) compared with the physicians who described that knowledge as rather or very bad (Table 4 and Table 5). Similar associations were observed for moderate level of knowledge and identification and notification of patients’ smoking status (OR = 3.2; *p* < 0.01; OR = 3.1; *p* < 0.05, respectively). Physicians who declared hospital as place of primary employment, compared to those in ambulatory care, were more likely (OR = 3.6; *p* < 0.01) to ask patients about smoking cigarettes when routinely collecting anamnesis (Table 4). Physicians with more years of professional experience were more likely to ask a patient about e-cigarette use (OR = 1.1; *p* < 0.05) (Table 5). Females (OR = 2.7; *p* < 0.05) as well as those who had ever (OR = 9.5; *p* < 0.05) and never tried e-cigarettes (OR = 12.2; *p* < 0.05) were more likely to write down in the medical documentation the patients’ cigarette smoking status comparing to males and current e-cigarette users (Table 4). Physicians trained in a nonsurgical specialty used minimal intervention on smoking cessation more often with patients who smoke cigarettes (OR = 2.7; *p* < 0.001) or use e-cigarette (OR = 1.9; *p* < 0.05), compared to those trained in surgical specialties (Table 4 and Table 5). Specialists more often (OR = 2.5; *p* < 0.05) referred e-cigarette users to smoking cessation clinics than physicians in training (Table 5). Physicians who had never tried cigarettes more often referred smokers or e-cigarette users to smoking cessation clinics, compared to physicians who had tried at least one cigarette (OR = 2.3; *p* < 0.05; OR = 3.1; *p* < 0.01, respectively) (Table 4 and Table 5).

## 4. Discussion

To the authors’ best knowledge, this is the first study correlating the knowledge, attitudes, and behaviors toward smoking cessation and vaping cessation interventions among physicians in Poland. The current study indicates that physicians in Poland declare a moderate level of knowledge about smoking cessation interventions. Self-declared level of knowledge about smoking cessation intervention shaped physicians’ behaviors toward cigarette and e-cigarette use by the patients. Also, physicians’ own smoking status determined smoking cessation intervention offered to patient who smoke or use e-cigarette. Our study revealed that physicians in Poland do not routinely provide smoking cessation intervention to their patients and most smoking cessation behaviors are limited to identification of the smokers. Never-smokers more often used minimal intervention on smoking cessation compared to those who had ever tried cigarettes or e-cigarettes. Despite the fact that, in Poland, e-cigarettes are classified as tobacco-related products [25], we observed significant differences between smoking cessation interventions for cigarette smokers and e-cigarette users. Smoking cessation activities were focused mostly on cigarette smoking and the dominant group of doctors does not ask patients about e-cigarettes. 

An analysis of tobacco use and smoking cessation practices (between 1987 and 2010) among physicians in developing countries shows that smoking prevalence was highest in Central and Eastern Europe (37%) and lowest in Asia (17.5%) [27]. However, in some European Union (EU) countries, the prevalence of tobacco use among physicians is equal to or even higher than its prevalence in the general population (28% in Italy; 34% in France; 40% in Greece) [39,40,41]. The high smoking prevalence among physicians might be explained by occupational stress, which is considered a key factor to addiction [42]. Moreover, this suggests an urgent need to develop smoking cessation promotion program at workplace of physicians [42,43]. In our study, the prevalence of tobacco use among physicians was 7.8%, which is lower than the prevalence of tobacco use in the general population of Poland (24%) [44]. The prevalence of tobacco use among physicians in our study is comparable to the 2014 studies of physicians in Estonia (6.7%) [45] and Spain (8.7%) [46]. Currently, there is a lack of epidemiological data on the prevalence of e-cigarette and heated tobacco use among physicians in the EU. In our study, 2% of physicians declared use of e-cigarette and an additional 2% reported current heated tobacco use, doubling prevalence in the general population (1%) [44], but lower than reported by medical students (2.9%) in Poland [47]. 

The current study indicates that physicians’ smoking status has a significant impact on smoking cessation interventions and influenced introduction of these interventions by the physicians. Similar results were observed in literature review by Abdullah et al. [27] on smoking cessation practices among physicians in developing countries. In the study by Abdullah et al., nonsmoker physicians more often questioned their patients about tobacco use than doctors who do smoke cigarettes [27]. 

Tobacco use increases the risk of multiple diseases, including coronary heart disease, chronic obstructive pulmonary disease, and cancers [1]. Due to the health burden of tobacco use, tobacco-related diseases concern the majority of medical specialties. In our study, 22% of physicians declared that they do not have contact with smoking patients in daily medical practice. Most of the physicians who neglected to mention smoking with their patients were pediatricians, which may suggest that they do not pay attention to smoking issues due to the age range of their patients. Nevertheless, the age of smoking initiation in Europe is decreasing [48], and younger people are reaching for cigarettes. Among teenagers, e-cigarettes are particularly popular [47,49]. In Poland, regular e-cigarette use is declared by every third (34%) student aged 15 to 19 [49]. Moreover, we can speculate that the group of physicians who declared they do not have contact with smoking patients in daily medical practice did not adequately address the patient’s smoking status, so did not mention any smoking cessation activities.

The current study indicates significant differences in smoking cessation interventions for patients smoking cigarettes compared to those using e-cigarettes. Two thirds of physicians regularly asked patients about cigarette smoking but only one-fifth (21.1%) of physicians regularly asked patients about e-cigarette use. Most of the physicians noted information about cigarette smoking in patients’ health records, wherein the information about e-cigarette was often missing. A systematic review of 35 papers on self-reported smoking cessation counseling by primary care physicians revealed that an average of 65% (range: 7–100%) of physicians assessed smoking status of their patients [50], which is comparable to the results of our study. The analysis of more than five years of electronic health records in Minneapolis (MN, US) demonstrated increasing documentation of e-cigarette use, however those notes were occasional and unstructured [51]. Introduction of electronic health records may increase the proportion of patients with recorded smoking status [52]. According to the National Tobacco Control Act in Poland, e-cigarettes are considered equivalent to conventional tobacco cigarettes [25]. The legal status of e-cigarettes may suggest that the use of e-cigarettes should be treated by physicians in the same way as cigarette smoking. However, national smoking cessation guidelines do not include e-cigarettes and the manner of addressing patients using e-cigarettes is not determined [26]. The highest proportion of physicians who asked about e-cigarette use was observed among specialists and doctors with the highest years of professional experience. We can hypothesize that the frequency of asking the patient about e-cigarette use was determined by the physician’s opinion, experience, and perception of e-cigarettes and their potential health effects. 

It is estimated that approximately 70% of smokers declare willingness to quit smoking [53]. However, studies performed in the US and Europe show that most smokers do not receive advice to quit smoking when visiting their physicians [30,54]. Moreover, most physicians do not arrange follow-up visits or assist patients with a cessation plan [27,55]. Similarly, our study finds that advising patients to quit smoking was not common behavior among the participating physicians. Minimal intervention on smoking cessation was regularly offered to smoking patients by 37.6% of physicians and 5% proposed referral to smoking cessation clinics. Never-smokers and physicians trained in nonsurgical specialties most actively offered smoking cessation interventions to smokers. Tobacco control authorities emphasize that tobacco dependence should be recognized as a lethal noncommunicable disease and be treated as a form of drug addiction [56,57]. However, in this study, physicians declared moderate knowledge of smoking cessation methods. Limited knowledge and skills regarding smoking cessation are the main reasons for physicians’ limited involvement with tobacco control efforts in European countries [27]. To increase physician engagement in tobacco control, there is need to develop training programs on evidence-based smoking cessation treatments. Moreover, there is need to develop tobacco control activities tailored to the needs of different population groups [58,59]. Smoking cessation courses should be considered a mandatory part of postgraduate medical education for physicians and allied healthcare professionals. 

This study has several limitations. First, it involved a small, select group of physicians attending courses at the School of Public Health, the Centre of Postgraduate Medical Education in Warsaw, Poland. The course is mandatory for all physicians undergoing specialty training in Poland and our voluntary study participants represented different regions and healthcare institutions from all over Poland. Second, smoking cessation interventions were assessed based on self-declared responses to the prepared questions. We are not able to verify whether the frequency of smoking cessation activities declared by doctors is reflected in the patients’ medical records. However, participants represented different medical units and the lack of a national electronic medical records system makes verification of patients’ smoking status almost impossible. Third, our study group included physicians from 52 different specialties, but more study is needed to assess the smoking cessation interventions in particular groups of medical specialists, especially general practitioners.

## 5. Conclusions

Among physicians in Poland, smoking cessation intervention was not common practice and most smoking cessation behaviors were limited to identification of smokers. There were differences in the tobacco cessation interventions offered to cigarette smokers and e-cigarette users. Tobacco cessation activities conducted by doctors focused mainly on tobacco use, with omission of e-cigarettes use. Physicians’ behaviors toward smoking cessation and vaping cessation interventions depended on the physicians’ smoking status and self-declared knowledge about smoking cessation methods. Education and training on smoking cessation and vaping cessation methods are needed to increase physicians’ involvement in tobacco control activities.

## Figures and Tables

**Table 1:** Subjects’ characteristics (n = 423).

	n (%)
Age, mean ± SD	32.0 ± 5.8
Sex	
Male	151 (35.7)
Female	272 (64.3)
Medical Education Level	
In training (during specialization)	361 (85.3)
Specialist	62 (14.7)
Specialty (n = 400)	
Surgical	110 (27.5)
Nonsurgical	290 (72.5)
Years of Professional Experience, mean ± SD	6.0 ± 5.4
Place of Primary Employment (practice type)	
Hospital	374 (88.4)
Ambulatory	49 (11.6)
Practice Location	
Rural	9 (2.1)
City up to 200,000 residents	123 (29.1)
City from 200,000 to 500,000 residents	67 (15.8)
City above 500,000 residents	224 (53.0)

SD: standard deviation.

**Table 2:** Prevalence of cigarette, e-cigarette, and heated tobacco use among physicians.

	Cigarette		E-Cigarette			Heated Tobacco		
	Ever Smoke % (95% CI)	Current Smoke % (95% CI)	Awareness % (95% CI)	Ever Use % (95% CI)	Current Use % (95% CI)	Awareness % (95% CI)	Ever Use % (95% CI)	Current Use % (95% CI)
Overall n = 423	71.2 (66.7–75.3)	7.8 (5.6–10.8)	99.8 (98.7–100.0)	22.2 (18.5–26.4)	1.9 (1.0–3.7)	42.6 (37.9–47.3)	8.5 (6.2–11.6)	1.9 (1.0–3.7)
Gender								
Male n = 151	78.8 (71.6–84.6)	10.6 (6.6–16.5)	100.0 (97.5–100.0)	33.8 (26.7–41.6)	2.7 (1.0–6.6)	57.0 (49.0–64.6)	13.3 (8.7–19.6)	3.3 (1.4–7.5)
Female n = 272	66.9 (61.1–72.2)	6.3 (3.9–9.8)	99.6 (97.9–99.9)	15.8 (12.0–20.6)	1.5 (0.6–3.7)	34.6 (29.2–40.4)	5.9 (3.7–9.3	1.1 (0.4–3.2)
*p*	0.01	0.1	0.5	<0.001	0.4	<0.001	0.01	0.1
Medical education level								
Residents n = 361	73.7 (68.9–78.0)	8.0 (5.7–11.3)	99.7 (98.5–100.0)	24.1 (20.0–28.8)	2.2 (1.1–4.3)	43.2 (38.2–48.4)	8.9 (6.4–12.3)	1.7 (0.8–3.6)
Specialists n = 62	56.5 (44.1–68.1)	6.5 (2.5–15.5)	100.0 (94.2–100.0)	11.3 (5.6–21.5)	0.0 (0.0–5.8)	38.7 (27.6–51.2)	6.5 (2.5–15.5)	3.2 (0.9–11.0)
*p*	0.006	0.7	0.7	0.03	0.2	0.5	0.5	0.4
Specialty								
Surgical n = 110	76.4 (67.6–83.3)	10.0 (5.7–17.0)	100.0 (96.6–100.0)	33.6 (25.5–42.9)	1.8 (0.5–6.4)	52.7 (43.5–61.8)	12.7 (7.7–20.2)	2.7 (0.9–7.7)
Nonsurgical n = 290	69.0 (63.4–74.0)	7.2 (4.8–10.8)	99.7 (98.1–99.9)	18.3 (14.3–23.1)	1.7 (0.7–4.0)	39.0 (33.5–44.7)	6.9 (4.5–10.4)	1.4 (0.5–3.5)
*p*	0.1	0.4	0.5	0.001	0.9	0.01	0.06	0.4

95%CI: 95% confidence interval; *p*: result of Chi-square test.

**Table 3:** Physicians’ self-reported behaviors toward cigarette or e-cigarette use by the patients (n=330).

	Physicians’ Self-Reported Behaviors toward Cigarette Smoking by the Patients % (95%CI)	Physicians’ Self-Reported Behaviors toward E-Cigarette Use by the Patients % (95%CI)	*p*
Do you ask a patient about smoking cigarettes or e-cigarette use when collecting anamnesis?			
Yes, in every case	66.7 (61.4–71.6)	21.1 (17.0–25.9)	<0.001
Yes, in more than half of the cases	22.0 (17.9–26.8)	11.9 (8.9–15.9)	
Yes, in less than half of the cases	9.8 (7.0–13.5)	20.2 (16.2–24.9)	
No, never	1.5 (0.7–3.5)	46.8 (41.5–52.2)	
Do you write down in the medical documentation the patients’ smoking status?			
Yes, in every case	78.7 (73.9–82.8)	32.2 (27.3–37.5)	<0.001
Yes, in more than half of the cases	12.5 (9.4–16.5)	9.9 (7.1–13.7)	
Yes, in less than half of the cases	6.4 (4.2–9.6)	11.5 (8.4–15.4)	
No, never	2.4 (1.2–4.7)	46.4 (41.1–51.9)	
Do you use minimal intervention on smoking cessation in patients who smoke?			
Yes, in every case	37.6 (32.5–43.0)	20.6 (16.5–25.3)	<0.001
Yes, in more than half of the cases	23.9 (19.6–28.8)	12.9 (9.7–17.0)	
Yes, in less than half of the cases	20.8 (16.8–25.5)	17.5 (13.8–22.0)	
No, never	17.7 (14.0–22.2)	49.0 (43.7–54.5)	
Do you suggest a referral to anti-smoking counseling to a patient who smokes?			
Yes, in every case	5.5 (3.5–8.5)	3.4 (1.9–5.9)	0.002
Yes, in more than half of the cases	8.8 (6.2–12.4)	4.9 (3.0–7.8)	
Yes, in less than half of the cases	16.5 (12.8–20.9)	11.3 (8.3–15.2)	
No, never	69.2 (64.0–74.0)	80.5 (75.9–84.4)	

95%CI: 95% confidence interval; *p*: result of linear-by-linear Association test.

**Table 4:** Associations between personal characteristics and self-reported behaviors toward cigarette smoking by the patients (n=330).

	Do You Ask a Patient about Smoking Cigarettes When Collecting Anamnesis?		Do You Write down in the Medical Documentation the Patients’ Cigarette Smoking Status?		Do You Use Minimal Intervention on Smoking Cessation in Patients Who Smoke Cigarettes?		Do You Suggest a Referral to Anti-Smoking Counseling to a Patient Who Smokes Cigarettes?	
	Unadjusted	Adjusted	Unadjusted	Adjusted	Unadjusted	Adjusted	Unadjusted	Adjusted
	Odds Ratio (95% CI)							
Gender								
Male	1.00 Ref.		1.00 Ref.	1.00 Ref.	1.00 Ref.		1.64 (0.88–3.07)	
Female	1.36 (0.68–2.73)		2.70 * (1.24–5.88)	2.70 * (1.17–6.23)	1.30 (0.82–2.06)		1.00 Ref.	
Age (years)	0.98 (0.93–1.03)		0.98 (0.93–1.04)		1.01 (0.97–1.04)		1.02 (0.97–1.07)	
Years of professional experience	0.99 (0.93–1.06)		0.98 (0.92–1.05)		1.00 (0.96–1.05)		1.00 (0.94–1.06)	
Medical education level								
Residents	1.00 Ref.		1.00 Ref.		1.00 Ref.		1.00 Ref.	
Specialists	1.27 (0.47–3.44)		1.74 (0.50–6.00)		1.73 (0.90–3.29)		1.74 (0.82–3.69)	
Specialty								
Surgical	1.42 (0.61–3.28)		1.00 Ref.		1.00 Ref.	1.00 Ref.	1.00 Ref.	
Nonsurgical	1.00 Ref.		1.19 (0.51–2.77)		2.69 *** (1.62–4.45)	2.68 *** (1.60–4.50)	1.19 (0.57–2.49)	
Place of primary employment								
Hospital	2.70 * (1.20–6.07)	3.61 ** (1.52–8.60)	1.74 (0.66–4.56)		1.07 (0.56–2.05)		1.00 Ref.	
Ambulatory	1.00 Ref.	1.00 Ref.	1.00 Ref.		1.00 Ref.		1.35 (0.58–3.13)	
Practice location								
Rural + City up to 200,000 residents	1.00 Ref.		1.00 Ref.		1.26 (0.76–2.07)		1.06 (0.54–2.07)	
City from 200,000 to 500,000 residents	2.15 (0.68–6.77)		0.62 (0.21–1.87)		1.15 (0.59–2.23)		0.67 (0.24–1.88)	
City above 500,000 residents	2.01 (0.96–4.19)		1.00 (0.41–2.39)		1.00 Ref.		1.00 Ref.	
Self-reported knowledge of smoking cessation counseling								
Very good or rather good	7.80 *** (2.54–23.96)	9.82 *** (3.06–31.49)	4.29 * (1.42–12.97)	6.55 ** (1.94–22.09)	2.88 ** (1.40–5.92)	3.27 ** (1.50–7.15)	4.39 * (1.25–15.40)	4.42 * (1.25–15.67)
Moderate	2.89 ** (1.28–6.52)	3.16 ** (1.37–7.30)	2.86 * (1.14–7.14)	3.09 * (1.18–8.12)	1.64 (0.85–3.18)	1.52 (0.75–3.12)	1.61 (0.45–5.78)	1.55 (0.43–5.59)
Rather bad or very bad	1.00 Ref.	1.00 Ref.	1.00 Ref.	1.00 Ref.	1.00 Ref.	1.00 Ref.	1.00 Ref.	1.00 Ref.
Cigarette smoking								
Current	1.00 Ref.		1.00 Ref.		1.00 Ref.		1.00 Ref. (current or ever) #	1.00 Ref. (current or ever) #
Ever	1.22 (0.39–3.84)		1.56 (0.49–4.98)		1.21 (0.54–2.73)			
Never	2.09 (0.56–7.80)		3.78 (0.87–16.41)		1.55 (0.64–3.72)		2.19 * (1.16–4.15)	2.31 * (1.20–4.46)
E-cigarette use								
Current	1.00 Ref.		1.00 Ref.	1.00 Ref.	1.00 Ref.		1.00 Ref. (current or ever) #	
Ever	2.04 (0.35–11.79)		4.28 * (1.01–21.58)	9.46 * (1.53–58.32)	1.50 (0.34–6.60)			
Never	2.94 (0.56–15.41)		7.90 ** (1.73–36.03)	12.24 * (2.20–67.93)	1.65 (0.40–6.81)		2.14 (0.87–5.28)	
Heated tobacco use								
Current or Ever #	1.87 (0.42–8.25)		1.49 (0.33–6.45)		1.00 Ref.		1.17 (0.42–3.22)	
Never	1.00 Ref.		1.00 Ref.		1.17 (0.55–2.49)		1.00 Ref.	

* *p* < 0.05; ** *p* < 0.01; *** *p* < 0.001; # combined categories were created taking into account small number of participants with positive responses.

**Table 5:** Associations between personal characteristics and self-reported behaviors toward e-cigarette use by the patients (n = 330).

	Do You Ask a Patient about E-Cigarette Use When Collecting Anamnesis?		Do You Write Down in the Medical Documentation the Patients’ E-Cigarette Use Status?		Do You Use Minimal Intervention on Smoking Cessation in Patients Who Use E-Cigarettes?		Do You Suggest a Referral to Anti-Smoking Counseling to a Patient Who Uses E-Cigarettes?	
	Unadjusted	Adjusted	Unadjusted	Adjusted	Unadjusted	Adjusted	Unadjusted	Adjusted
Odds Ratio (95% CI)								
Gender								
Male	1.49 (0.93–2.83)		1.08 (0.69–1.71)		1.00 Ref.		1.98(0.90–4.38)	
Female	1.00 Ref.		1.00 Ref.		1.45 (0.89–2.36)		1.00 Ref.	
Age (years)	1.03 (0.99–1.07)		1.02 (0.99–1.06)	1.02 (0.99–1.06)		1.04 (0.99–1.10)		
Years of professional experience	1.05 * (1.01–1.09)	1.05 * (1.01–1.09)	1.03 (0.99–1.08)	1.03 (0.99–1.08)		1.00 (0.93–1.08)		
Medical education level								
Residents	1.00 Ref.		1.00 Ref.		1.00 Ref.		1.00 Ref.	
Specialists	1.78 (0.98–3.23)		1.33 (0.73–2.43)		2.20 (1.21–4.01)		2.92 * (1.23–6.94)	2.49 *(1.00–6.22)
Specialty								
Surgical	1.24 (0.74–2.08)		1.30 (0.79–2.14)		1.00 Ref.	1.00 Ref.	1.00 Ref.	
Nonsurgical	1.00 Ref.		1.00 Ref.		1.94* (1.10–3.40)	1.93 * (1.08–3.43)	1.15 (0.43–3.04)	
Place of primary employment								
Hospital	1.00 Ref.		1.09 (0.56–2.09)		1.23 (0.61–2.47)		1.00 Ref.	
Ambulatory	1.26 (0.66–2.43)		1.00 Ref.		1.00 Ref.		1.48 (0.53–4.15)	
Practice location								
Rural + City up to 200,000 residents	1.00 Ref.		1.00 Ref.		1.05 (0.63–1.76)		1.11 (0.48–2.61)	
City from 200,000 to 500,000 residents	1.90 (0.93–3.89)		1.63 (0.82–3.25)		1.40 (0.72–2.71)		0.74 (0.20–2.71)	
City above 500,000 residents	1.42 (0.84–2.40)		1.21 (0.74–1.99)		1.00 Ref.		1.00 Ref.	
Self-reported knowledge of smoking cessation counseling								
Very good or rather good	2.77 * (1.27–6.04)	2.37 * (1.07–5.25)	2.35 * (1.12–4.94)	2.35 * (1.12–4.94)	2.15 * (1.00–4.63)	2.70 * (1.15–6.32)	8.13 * (1.04–63.53)	8.00 * (1.01–63.45)
Moderate	1.13 (0.53–2.43)	1.00 (0.46–2.18)	1.33 (0.66–2.71)	1.33 (0.66–2.71)	1.11 (0.53–2.33)	1.24 (0.54–2.84)	2.40 (0.29–19.60)	2.14 (0.26–17.77)
Rather bad or very bad	1.00 Ref.	1.00 Ref.	1.00 Ref.	1.00 Ref.	1.00 Ref.	1.00 Ref.	1.00 Ref.	1.00 Ref.
Cigarette smoking								
Current	1.05 (0.42–2.63)		1.00 Ref.		1.00 Ref.		1.00 Ref.(current or ever) #	1.00 Ref. (current or ever) #
Ever	1.07 (0.63–1.81)		1.17 (0.51–2.72)		0.88 (0.38–2.08)			
Never	1.00 Ref.		1.19 (0.49–2.93)		1.33 (0.54–3.31)		3.13 ** (1.41–6.98)	3.06 ** (1.32–7.09)
E-cigarette use								
Current	1.00 Ref.		1.00 Ref.		1.00 Ref.		1.00 Ref. (current or ever) #	
Ever	1.43 (0.26–7.75)		1.46 (0.26–8.19)		1.24 (0.23–6.74)			
Never	1.53 (0.30–7.77)		1.95 (0.37–10.31)		1.60 (0.31–8.14)		1.71 (0.57–5.13)	
Heated tobacco use								
Current or Ever #	2.02 (0.96–4.28)		1.91 (0.89–4.10)		1.11 (0.51–2.41)		1.76 (0.57–5.50)	
Never	1.00 Ref.		1.00 Ref.		1.00 Ref.		1.00 Ref.	

* *p* < 0.05; ** *p* < 0.01; # combined categories were created taking into account small number of participants with positive responses

## References

[1] GBD 2015 Tobacco Collaborators (2017). Smoking prevalence and attributable disease burden in 195 countries and territories, 1990–2015: A systematic analysis from the Global Burden of Disease Study 2015. Lancet.

[2] World Health Organization WHO Report on the Global Tobacco Epidemic, 2008 The MPOWER Package. https://www.who.int/tobacco/mpower/mpower_report_full_2008.pdf.

[3] Hammond D., Fong G.T., McNeill A., Borland R., Cummings K.M. (2006). Effectiveness of cigarette warning labels in informing smokers about the risks of smoking: Findings from the International Tobacco Control (ITC) Four Country Survey. Tob. Control.

[4] Saraf D.S., Mehrotra R., Chandan K., Sinha D.N., Yadav A. (2018). A review of trade practices of smokeless tobacco products in terms of prohibition on sale, manufacturing & importation in Framework Convention on Tobacco Control ratified Parties. Indian J. Med. Res..

[5] Hoffman S.J., Mammone J., Rogers Van Katwyk S., Sritharan L., Tran M., Al-Khateeb S., Grjibovski A., Gunn E., Kamali-Anaraki S., Li B. (2019). Cigarette consumption estimates for 71 countries from 1970 to 2015: Systematic collection of comparable data to facilitate quasi-experimental evaluations of national and global tobacco control interventions. BMJ.

[6] World Health Organization (2003). Framework Convention on Tobacco Control.

[7] Ngo A., Cheng K.W., Chaloupka F.J., Shang C. (2017). The effect of MPOWER scores on cigarette smoking prevalence and consumption. Prev. Med..

[8] Levy D.T., Li Y., Yuan Z. (2019). Impact of nations meeting the MPOWER targets between 2014 and 2016: An update. Tob. Control.

[9] Wilson L.M., Avila Tang E., Chander G., Hutton H.E., Odelola O.A., Elf J.L., Heckman-Stoddard B.M., Bass E.B., Little E.A., Haberl E.B. (2012). Impact of tobacco control interventions on smoking initiation, cessation, and prevalence: A systematic review. J. Environ. Public Health.

[10] Stead L.F., Buitrago D., Preciado N., Sanchez G., Hartmann-Boyce J., Lancaster T. (2012). Physician advice for smoking cessation. Cochrane Database Syst. Rev..

[11] World Health Organization The Role of Health Professionals in Tobacco Control. https://www.who.int/tobacco/resources/publications/wntd/2005/bookletfinal_20april.pdf.

[12] Carson K.V., Verbiest M.E., Crone M.R., Brinn M.P., Esterman A.J., Assendelft W.J., Smith B.J. (2012). Training health professionals in smoking cessation. Cochrane Database Syst. Rev..

[13] Chapman S. (1993). The role of doctors in promoting smoking cessation. BMJ.

[14] Jassem J., Przewoźniak K., Zatoński W. (2014). Tobacco control in Poland-successes and challenges. Transl. Lung Cancer Res..

[15] Verbiest M., Brakema E., van der Kleij R., Sheals K., Allistone G., Williams S., McEwen A., Chavannes N. (2017). National guidelines for smoking cessation in primary care: A literature review and evidence analysis. NPJ. Prim. Care. Respir. Med..

[16] Jankowski P., Kawecka-Jaszcz K., Kopeć G., Podolec J., Pająk A., Sarnecka A., Zdrojewski T., Czarnecka D., Małecki M., Nowicka G. (2017). Polish Forum for Prevention Guidelines on Smoking: Update 2017. Kardiol. Pol..

[17] Kawecka-Jaszcz K., Jankowski P., Podolec P., Kopeć G., Naruszewicz M., Opala G., Godycki-Cwirko M., Kozek E., Stańczyk J., Undas A. (2008). Polish forum for prevention guidelines on smoking. Kardiol. Pol..

[18] National Institute for Health and Clinical Excellence Stop Smoking Interventions and Services. https://www.nice.org.uk/guidance/ng92/chapter/Recommendations#advice-on-ecigarettes.

[19] Piné-Abata H., McNeill A., Murray R., Bitton A., Rigotti N., Raw M. (2013). A survey of tobacco dependence treatment services in 121 countries. Addiction.

[20] Janik-Koncewicz K., Zatoński M., Herbeć A., Zatoński W.A. (2017). The role of the Health Promotion Foundation in building capacity to treat tobacco dependence in Poland: Past, present and future. J. Health Inequal..

[21] Marie Skłodowska-Curie Memorial Cancer Centre Training Programmes. https://szkoleniazut.coi.pl.

[22] World Medical Association WMA Statement on Electronic Cigarettes and Other Electronic Nicotine Delivery Systems. https://www.wma.net/policies-post/wma-statement-on-electronic-cigarettes-and-other-electronic-nicotine-delivery-systems/.

[23] Bhatnagar A., Whitsel L.P., Ribisl K.M., Bullen C., Chaloupka F., Piano M.R., Robertson R.M., McAuley T., Goff D., Benowitz N. (2014). Electronic cigarettes: A policy statement from the American Heart Association. Circulation.

[24] European Respiratory Society, ERS Tobacco Control Committee ERS Position Paper on Tobacco Harm Reduction. https://ers.app.box.com/v/ERSTCC-Harm-Reduction-Position.

[25] Parliament of the Republic of Poland Act from 22 July 2016 on Amending the Act on Protection of Health against the Consequences of the Use of Tobacco and Tobacco Products in Poland. Journal of Laws from 24 August 2016 (Item 1331). http://prawo.sejm.gov.pl/isap.nsf/DocDetails.xsp?id=WDU20160001331.

[26] Zgliczyński W.S., Jankowski M., Rostkowska O., Gujski M., Wierzba W., Pinkas J. (2019). Knowledge and beliefs of e-cigarettes among physicians in Poland. Med. Sci. Monit..

[27] Abdullah A.S., Stillman F.A., Yang L., Luo H., Zhang Z., Samet J.M. (2013). Tobacco use and smoking cessation practices among physicians in developing countries: A literature review (1987–2010). Int. J. Environ. Res. Public Health.

[28] Ellerbeck E.F., Ahluwalia J.S., Jolicoeur D.G., Gladden J., Mosier M.C. (2001). Direct observation of smoking cessation activities in primary care practice. J. Fam. Pract..

[29] Coleman T., Wynn A., Barrett S., Wilson A. (2003). Discussion of NRT and other antismoking interventions in UK general practitioners’ routine consultations. Nicotine Tob. Res..

[30] Stead M., Angus K., Holme I., Cohen D., Tait G. (2009). Factors influencing European GPs’ engagement in smoking cessation: A multi-country literature review. Br. J. Gen. Pract..

[31] Van Eerd E.A.M., BechRisør M., Spigt M., Godycki-Cwirko M., Andreeva E., Francis N., Wollny A., Melbye H., van Schayck O., Kotz D. (2017). Why do physicians lack engagement with smoking cessation treatment in their COPD patients? A multinational qualitative study. NPJ. Prim. Care Respir. Med..

[32] Jiang Y., Ong M.K., Tong E.K., Yang Y., Nan Y., Gan Q., Hu T.W. (2007). Chinese physicians and their smoking knowledge, attitudes, and practices. Am. J. Prev. Med..

[33] Poanta L.I., Zdrenghea D., Albu A. (2006). Psychometric evaluation of Romanian version of job content questionnaire in physicians. Rom. J. Int. Med..

[34] Reile R., Pärna K. (2018). Do physicians address their patients’ smoking behavior? Results from a nationwide survey among physicians in Estonia. Public Health.

[35] Kanchustambham V., Saladi S., Rodrigues J., Fernandes H., Patolia S., Santosh S. (2017). The knowledge, concerns, and healthcare practices among physicians regarding electronic cigarettes. J. Community Hosp. Intern. Med. Perspect..

[36] Kollath-Cattano C., Dorman T., Albano A.W., Jindal M., Strayer S.M., Thrasher J.F. (2019). E-cigarettes and the clinical encounter: Physician perspectives on e-cigarette safety, effectiveness, and patient educational needs. J. Eval. Clin. Pract..

[37] Pepper J.K., McRee A.L., Gilkey M.B. (2014). Healthcare providers’ beliefs and attitudes about electronic cigarettes and preventive counseling for adolescent patients. J. Adolesc. Health.

[38] Kandra K.L., Ranney L.M., Lee J.G.L., Goldstein A.O. (2014). Physicians’ attitudes and use of e-cigarettes as cessation devices, North Carolina 2013. PLoS ONE.

[39] Chapman S. (1995). Doctors who smoke. BMJ.

[40] Adriaanse H., van Reek J. (1989). Physicians’ smoking and its exemplary effect. Scand. J. Prim. Health Care.

[41] Sotiropoulos A., Gikas A., Spanou E., Dimitrelos D., Karakostas F., Skliros E., Apostolou O., Politakis P., Pappas S. (2007). Smoking habits and associated factors among Greek physicians. Public Health.

[42] Juranić B., Rakošec Ž., Jakab J., Mikšić Š., Vuletić S., Ivandić M., Blažević I. (2017). Prevalence, habits and personal attitudes towards smoking among health care professionals. J. Occup. Med. Toxicol..

[43] Ficarra M.G., Gualano M.R., Capizzi S., Siliquini R., Liguori G., Manzoli L., Briziarelli L., Parlato A., Cuccurullo P., Bucci R. (2011). Tobacco use prevalence, knowledge and attitudes among Italian hospital healthcare professionals. Eur. J. Public Health.

[44] Kantar Public for Chief Sanitary Inspectorate A Report from a Nationwide Survey on Attitudes Towards Smoking. https://gis.gov.pl/wp-content/uploads/2018/04/Postawy-Polak%C3%B3w-do-palenia-tytoniu-Raport-2017.pdf.

[45] Raag M., Pärna K. (2018). Cigarette smoking and smoking-attributable diseases among Estonian physicians: A cross-sectional study. BMC Public Health.

[46] Jiménez-Ruiz C.A., Riesco Miranda J.A., Ramos Pinedo A., de Higes Martinez E., Marquez F.L., PalomoCobos L., Solano Reina S., de GrandaOrive J.I., de Lucas Ramos P. (2015). Prevalence of and Attitudes towards Smoking among Spanish Health Professionals. Respiration.

[47] Brożek G.M., Jankowski M., Lawson J.A., Shpakou A., Poznański M., Zielonka T.M., Klimatckaia L., Loginovich Y., Rachel M., Gereová J. (2019). The Prevalence of Cigarette and E-cigarette Smoking Among Students in Central and Eastern Europe—Results of the YUPESS Study. Int. J. Environ. Res. Public Health.

[48] Marcon A., Pesce G., Calciano L., Bellisario V., Dharmage S.C., Garcia-Aymerich J., Gislasson T., Heinrich J., Holm M., Janson C. (2018). Trends in smoking initiation in Europe over 40 years: A retrospective cohort study. PLoS ONE.

[49] Smith D.M., Gawron M., Balwicki L., Sobczak A., Matynia M., Goniewicz M.L. (2019). Exclusive versus dual use of tobacco and electronic cigarettes among adolescents in Poland, 2010–2016. Addict. Behav..

[50] Bartsch A.L., Härter M., Niedrich J., Brütt A.L., Buchholz A. (2016). A Systematic Literature Review of Self-Reported Smoking Cessation Counseling by Primary Care Physicians. PLoS ONE.

[51] Winden T.J., Chen E.S., Wang Y., Sarkar I.N., Carter E.W., Melton G.B. (2015). Towards the Standardized Documentation of E-Cigarette Use in the Electronic Health Record for Population Health Surveillance and Research. AMIA Jt. Summits Transl. Sci. Proc..

[52] Wu C.Y., Chang C.K., Robson D., Jackson R., Chen S.J., Hayes R.D., Stewart R. (2013). Evaluation of smoking status identification using electronic health records and open-text information in a large mental health case register. PLoS ONE.

[53] Fagerstrom K., Boyle P., Kunze M., Zatonski W. (2001). The anti-smoking climate in EU countries and Poland. Lung Cancer.

[54] Caplan L., Stout C., Blumenthal D.S. (2011). Training physicians to do office-based smoking cessation increases adherence to PHS guidelines. J. Community Health.

[55] Gunes G., Karaoglu L., Genc M.F., Pehlivan E., Egri M. (2005). University hospital physicians’ attitudes and practices for smoking cessation counseling in Malatya, Turkey. Patient Educ. Couns..

[56] Xiao D., Wang C. (2019). Tobacco dependence should be recognised as a lethal non-communicable disease. BMJ.

[57] Kmietowicz Z. (2000). Doctors told to treat nicotine addiction as a disease. BMJ.

[58] Kaleta D., Polanska K., Bak-Romaniszyn L., Wojtysiak P. (2016). Perceived Relative Harm of Selected Cigarettes and Non-Cigarette Tobacco Products—A Study of Young People from a Socio-Economically Disadvantaged Rural Area in Poland. Int. J. Environ. Res. Public Health.

[59] Kaleta D., Kalucka S., Szatko F., Makowiec-Dąbrowska T. (2017). Prevalence and Correlates of Physical Inactivity during Leisure-Time and Commuting among Beneficiaries of Government Welfare Assistance in Poland. Int. J. Environ. Res. Public Health.

